# Variational Quantum Algorithm Applied to Collision Avoidance of Unmanned Aerial Vehicles

**DOI:** 10.3390/e24111685

**Published:** 2022-11-18

**Authors:** Zhaolong Huang, Qiting Li, Junling Zhao, Meimei Song

**Affiliations:** 1 College of Science, Tianjin University of Technology, Tianjin 300384, China; 2 R & D Department, China Academy of Launch Vehicle Technology, Beijing 100076, China

**Keywords:** collision avoidance of UAVs, variational quantum algorithms, variational quantum eigensolver, quantum approximate optimization algorithm, conditional value-at-risk

## Abstract

Mission planning for multiple unmanned aerial vehicles (UAVs) is a complex problem that is expected to be solved by quantum computing. With the increasing application of UAVs, the demand for efficient conflict management strategies to ensure airspace safety continues to increase. In the era of noisy intermediate-scale quantum (NISQ) devices, variational quantum algorithms (VQA) for optimizing parameterized quantum circuits with the help of classical optimizers are currently one of the most promising strategies to gain quantum advantage. In this paper, we propose a mathematical model for the UAV collision avoidance problem that maps the collision avoidance problem to a quadratic unconstrained binary optimization (QUBO) problem. The problem is formulated as an Ising Hamiltonian, then the ground state is solved using two kinds of VQAs: the variational quantum eigensolver (VQE) and the quantum approximate optimization algorithm (QAOA). We select conditional value-at-risk (CVaR) to further promote the performance of our model. Four examples are given to validate that with our method the probability of obtaining a feasible solution can exceed 90% based on appropriate parameters, and our method can enhance the efficiency of a UAVs’ collision avoidance model.

## 1. Introduction

The emergence of UAVs has dramatically changed the way people use transportation [1], such as aerial logistics networks and short- and medium-range manned transportation at low altitude. UAVs have the characteristics of a small safety risk factor and low cost. Therefore, in the foreseeable future, the frequency of UAV use will rise, and the workload of low-altitude air traffic flow management (ATFM) will also increase accordingly. Deploying UAVs on a large scale faces an issue of collision avoidance problem of UAVs. Therefore, it is necessary to build an algorithm for unmanned traffic management (UTM) [2]. We need to determine the UAVs’ collision avoidance actions, including takeoff delays and local maneuvers, that the aircraft needs to implement in real time according to the potential conflicts of different aircraft routes, and avoid conflicts while ensuring a low cost of collision avoidance. Mathematically, this problem is an optimization problem to avoid collisions at minimum cost. With the expansion of the problem scale and the increase of constraints, the required computing resources also increase exponentially, which leads to increasing difficulty of solving optimization problems.

As quantum computers have continued to advance in recent decades, researchers have begun to use quantum computers to find solutions to optimization problems [3,4,5]. Quantum computers have the capability of parallel computing and considerable computing advantages over classical computers. In some specific tasks, they have even surpassed the current most advanced classical computers [6,7,8]. Current quantum computers are noisy intermediate-scale quantum (NISQ) computers [9]. Although the most advanced quantum computers have reached fifty to hundreds of qubits, they are still unable to achieve fault tolerance, hence fault-tolerant quantum computers may not be available in the short term. VQA combines the advantages of quantum and classical computing by using parametric quantum circuits running on a quantum computer followed by parametric optimization on a classical optimizer [10]. Utilizing classical computers, VQA, not only compensates for the shortage of quantum bits and pushes the complexity to classical computers, but running only short-depth quantum circuits is an extremely effective strategy to reduce errors in NISQ devices.

Given that VQA, with promising properties, can support a form of quantum advantage [11,12], and considerable progress have been made in its experimental realization [13], we select popular VQA, such as the variational quantum eigensolver (VQE) [14] and the quantum approximate optimization algorithm (QAOA) [5], to solve our collision avoidance problem. Furthermore, in order to enhance the efficiency of VQA, we also introduce the recently proposed conditional value-at-risk (CVaR) as an aggregation function to optimize the original cost function and compare the performance with the original VQA [4].

When solving optimization problems with quantum computers, the optimization problem is often formulated as a quadratic unconstrained binary optimization (QUBO) problem [15]. In the transportation domain, researchers have used quantum computing to solve air traffic management problems. A kind of air traffic management (ATM) problem has been converted to a QUBO problem and handled by a quantum computer [3]. Additionally, quantum computing is also being used for railway conflict management problems [16].

In the field of public transportation, UAVs are gaining attention as a promising means of transportation, and they have become one of the main available options for the transportation of medical supplies [17]. Internationally, there are already many mature and well established applications for drone transport, such as the East African medical drone transport [18], Amazon’s PrimeAir drone delivery service, etc. In 2021, Sumitomo Japan developed thousands of flight paths for drones through quantum computing for Urban Air Mobility [19]. In order to maintain the efficiency and safety of the both goods and people, the National Aeronautics and Space Administration (NASA) proposed the concept of Urban Air Mobility (UAM) in 2017 [20].

UAV transportation missions require huge numbers of UAVs to collaborate, which involves the route planning of multiple UAVs that include collision avoidance, task allocation, and so on. In this paper, we study the collision avoidance problem of UAVs based on VQA. We consider not only spatio-temporal constraints, but also the necessary factors such as preparation time, early takeoff time, and heterogeneity among multiple UAVs that should be accounted in practical missions. Inspired by the QUBO method in [3], we mathematically model the UAV collision avoidance problem in Section 2 through considering the collision avoidance by delaying the takeoff time of the aircraft and transforming the problem into a QUBO problem after discretization. Following that, we discuss the Ising model for the QUBO problem and related concepts of the variational quantum algorithm in Section 3. In Section 4, we present the test results of the four examples. Finally, we present a summary and outlook of the article.

Based on the particulars of the actual UAV flight, we considered the following conditions:

- Spatio-temporal conditions. For the objective requirement of the aircraft to ensure safety of flight, the distance between any two aircraft at the same point in time must be greater than a threshold value. From the standpoint of flight tasks, this requires us to adjust the trajectory of the aircraft or change the time of arrival at the conflict point when a conflict occurs. If we put it in mathematical perspective, it means that we must ensure that, for at least one of the two, the spatial function *A* and the temporal function *T*, should be bigger than 0.
- Preparation condition. Because of the special features of UAVs, the aircraft require preparation before takeoff. Nevertheless, the preparation time needed before takeoff is different for various types of aircraft, so we set a non-negative extra time for each aircraft, which is named pre-takeoff preparation time.
- Early takeoff condition. Unlike civil flights carrying people, in the mission of the UAVs we allow aircraft to take off earlier than scheduled departure time in order to avoid conflicts. This means that the departure delay time of the aircraft can not only be positive but also be negative.
- Heterogeneity condition. A UAV formation has different types and different models of aircraft that are required to perform different missions. For the priority of the aircraft resulting from the importance of missions to be performed or the urgency of tasks to be executed, we assign corresponding weights to each aircraft.

## 2. UAV Collision Avoidance Theory

The collision avoidance problem of UAVs is a complex problem and an important part of route planning. The input of the problem is a series of aircraft trajectories. For the purpose of saving flight cost, we tend to choose the optimal flight trajectory that has the lowest flight cost and minimum flight time when planning the route for the aircraft. This leads to the possibility that the optimal flight paths chosen by different aircraft will overlap with one another, thus increasing the probability of aircraft collisions. In this paper, assuming that the trajectories are known, we focus on the collision elimination by adjusting the takeoff time.

In the collision avoidance problem, the most common solution strategy is to change course or speed, but this also inevitably increases the cost of flight. However, the easiest way to de-conflict trajectory conflicts while taking into account flight costs is to delay the departure of aircraft having conflicting routes. When the delay time is short, there is hardly any negative impact on the completion of most tasks. This is the strategy we will utilize and analyze in this article.

### 2.1. Problem Description

In this research, it is assumed that delaying the takeoff time of a certain aircraft will not introduce new conflicts, and the input trajectory is $A_{i}=\left(A_{i,t_{i}}\right)$, where $A=\left\{i|i\in [1,F]\right\}$ represents the set of aircraft, $A_{i,t}$ represents the position of the aircraft *i* at time *t* (including its longitude, latitude, and altitude), $t_{i}\in \left[\tau_{i,0},\tau_{i,1}\right]$ is the flight time of aircraft *i*, and $\tau_{i,0}$ and $\tau_{i,1}$ are the takeoff time and landing time of *i*, respectively.

Let the delay when the aircraft *i* arrives at the point $A_{i,t_{i}}$ be $D_{i,t_{i}}$ and the maximum and minimum delay allowed is, respectively, $D_{i,t_{i}}^{\max}$ and $D_{i,t_{i}}^{\min}$, then the total delay of all aircraft is

(1) $$D=\sum_{i=1}^{F}D_{i,\tau_{i,1}}.$$

The waypoint $A_{i,t_{i}},A_{j,t_{j}}$ of a pair of aircraft (i,j) will not conflict if and only if their space and time functions are greater than their respective separation criteria $\Delta_{a}$ and $\Delta_{t}$, where $\Delta_{a}$ and $\Delta_{t}$ are non-negative real numbers. For example, the position separation criteria $\Delta_{a}$ can be set to 10 m, and the time separation $\Delta_{t}$ can be set to 2 s. In the research on the collision avoidance of UAVs, the following conditions should be met:

(2) max{A,T}>0,

where

(3) $$A=∥A_{i,t_{i}}-A_{j,t_{j}}∥-\Delta_{a},$$

(4) $$T=∥\left(t_{i}+D_{i,t_{i}}\right)-\left(t_{j}+D_{j,t_{j}}\right)∥-\Delta_{t}.$$

Consider the values of *A* and *T*, if (i,j) exists a collision, then there must be $A_{i,t_{i}},A_{j,t_{j}}$ such that A<0 and T<0. By Equation (4) we can find that, if T<0, then

(5) $$\left|t_{i}-t_{j}\right|-\left|D_{j,t_{j}}-D_{i,t_{i}}\right|\leq \left|\left(t_{i}-t_{j}\right)-\left(D_{j,t_{j}}-D_{i,t_{i}}\right)\right|<\Delta_{t},$$

which implies that

(6) $$\left|D_{j,t_{j}}-D_{i,t_{i}}\right|+\Delta_{t}>\left|t_{i}-t_{j}\right|.$$

It can be seen that $\left(D_{i,t_{i}},D_{j,t_{j}}\right)\in \left[D_{i,t_{i}}^{\min},D_{i,t_{i}}^{\max}\right]\times \left[D_{j,t_{j}}^{\min},D_{j,t_{j}}^{\max}\right]$, i.e.,

(7) $$\begin{array}{c} D_{i,t_{i}}^{\min}\leq D_{i,t_{i}}\leq D_{i,t_{i}}^{\max},\\ D_{j,t_{j}}^{\min}\leq D_{j,t_{j}}\leq D_{j,t_{j}}^{\max}. \end{array}$$

It follows from Equation (7) that

(8) $$D_{i,t_{i}}^{\min}-D_{j,t_{j}}^{\max}\leq D_{i,t_{i}}-D_{j,t_{j}}\leq D_{i,t_{i}}^{\max}-D_{j,t_{j}}^{\min}.$$

Combining Equations (6) and (8) yields

(9) $$\max\left\{|D_{i,t_{i}}^{\max}-D_{j,t_{j}}^{\min}\left|,\right|D_{j,t_{j}}^{\max}-D_{i,t_{i}}^{\min}|\right\}+\Delta_{t}>\left|t_{i}-t_{j}\right|,$$

and it is clear that there exists $\left(D_{i,t_{i}},D_{j,t_{j}}\right)$ making T<0, so the trajectories of (i,j) have the risk of conflict. Assuming that the closest conflict point of the trajectory distance of (i,j) is *M*, then there must be other potential conflict points near *M*.

**Figure 1:**
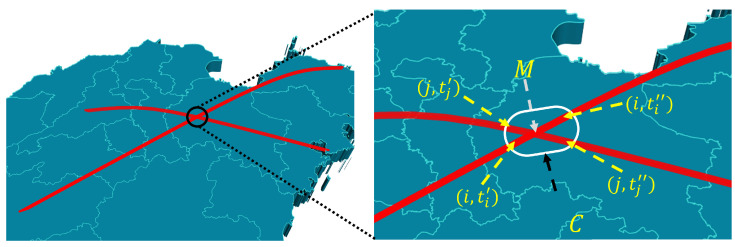
Schematic diagram of a pair of conflicting routes.

As shown in Figure 1, in the adjacent area *C* of *M*, we have A<0. Thus, if $A_{i,t_{i}^{\prime }}$ conflicts with $A_{j,t_{j}^{\prime }}$, and $A_{i,t_{i}^{\prime \prime }}$ also conflicts with $A_{j,t_{j}^{\prime \prime }}$, then for any $t_{i}\in \left[\min\left\{t_{i}^{\prime },t_{i}^{\prime \prime }\right\},\max\left\{t_{i}^{\prime },t_{i}^{\prime \prime }\right\}\right]$, there exists $t_{j}\in \left[\min\left\{t_{j}^{\prime },t_{j}^{\prime \prime }\right\},\max\left\{t_{j}^{\prime },t_{j}^{\prime \prime }\right\}\right]$ for which $A_{i,t_{i}}$ conflicts with $A_{j,t_{j}}$. These conflicts can be called conflicts derived from point *M*, and we can express the whole of the conflicts derived from point *M* as $C_{m}$. Similarly, the set of all aircraft collisions can be expressed as the set of mutually disjoint $C_{m}$,

(10) $$C=⋃C_{m},$$

We set the aircraft related to the conflict *m* as $R_{m}=\left\{i|i\in C_{m}\right\}$, then the set of conflicts related to the aircraft *i* is $M_{i}=\left\{m|i\in R_{m}\right\}$. Let $d_{i,m}$ be the scheduling delay of aircraft *i*, which is the delay incurred to prevent the collision *m*; $d_{i,p}$ is the pre-takeoff preparation time of aircraft *i*. This means that the total delay before aircraft *i* encounters collision *m* is

(11) $$\begin{array}{cc} D_{i,m} & =d_{i,p}+\sum_{m^{\prime }\in M_{i,m}}d_{i,m^{\prime }},\\ M_{i,m} & =\left\{m^{\prime }<m|m^{\prime }\in M_{i}\right\}. \end{array}$$

Then, for the conflict *m* between aircraft *i* and *j*, the total time pair satisfying the condition T<0 can be written as

(12) $$T_{m}=\left\{\left(t_{i},t_{j}\right)|\left\{\left(i,t_{i}\right),\left(j,t_{j}\right)\right\}\in C_{m},i<j\right\},$$

where $t_{i}$ and $t_{j}$ denote times as non-negative real numbers. We can obtain a range $B_{m}$ from $T_{m}$,

(13) $$\begin{array}{cc} B_{m} & =\underset{\left(t_{i},t_{j}\right)\in T_{m}}{⋃}\left(-\Delta_{t}+t_{j}-t_{i},\Delta_{t}+t_{j}-t_{i}\right),\\ \; & =\left[1-\Delta_{t}+\min_{\left(t_{i},t_{j}\right)\in T_{m}}\left(t_{j}-t_{i}\right),\Delta_{t}-1+\max_{\left(t_{i},t_{j}\right)\in T_{m}}\left(t_{j}-t_{i}\right)\right]. \end{array}$$

If it is satisfied that $D_{m}=D_{i,m}-D_{j,m}\notin B_{m}$, which is equivalent to potential conflicts being avoided. Therefore, we can express the total delay as

(14) $$D=\sum_{i=1}^{F}\left(d_{i,p}+\sum_{m\in M_{i}}d_{i,m}\right).$$

In order to reduce unnecessary calculation costs, we can remove the aircraft with no conflicts when calculating the delay time, which means we only need to re-calculate the aircraft $i\in \left[1,N_{c}\right]\left(N_{c}\leq F\right)$ with conflict risk. We want to minimize the value of *D* while eliminating collisions through optimization. At the same time, we only consider the conflict avoidance by delaying the departure time of the aircraft, which means that for aircraft *i*, we can use $d_{i}$ to denote $\sum_{m\in M_{i}}d_{i,m}$, namely, the scheduling delay time of aircraft *i*. Thus, the total delay can be simplified as

(15) $$D=\sum_{i=1}^{N_{c}}D_{i},$$

where $D_{i}=d_{i,p}+d_{i}$ is the delay sum of aircraft *i*. The configuration space of the problem is $D=\left\{D_{i}|i\in \left[1,N_{c}\right]\right\}$. Taking into account the importance of missions or the urgency of tasks, we assign weights to the delays of each aircraft, and the above formula can be transformed into

(16) $$D=\sum_{i=1}^{N_{c}}f\left(i\right)\cdot D_{i}.$$

### 2.2. Mapping Problem to QUBO

We first encode the configuration space D as a series of binary variables, and then solve the collision avoidance problem with quantum computing. To achieve this, we must first discretize the delay time allowed by the aircraft. Let $\Delta_{d}$ be the discretization step size of the delay. Then $d_{i}^{\max}=N_{i}^{\max}\cdot \Delta_{d}$ is the maximum scheduling delay time allowed by aircraft *i*, so $d_{i}\in \left\{\Delta_{d}l|l\in \left[0,N_{i}^{\max}\right]\right\}$. At the same time, in some cases, we can also avoid conflicts by advancing the departure time of the aircraft, that is, the minimum scheduling delay time $d_{i}^{\min}=N_{i}^{\min}\cdot \Delta_{d}<0$, therefore $d_{i}\in \left\{l\Delta_{d}|l\in \left[N_{i}^{\min},N_{i}^{\max}\right]\right\}$. Similarly, $d_{i,p}$ can also be expressed as $d_{i,p}=p_{i}\cdot \Delta_{d}$, then $D_{i}=\ell \Delta_{d},\ell \in \left[p_{i}+N_{i}^{\min},p_{i}+N_{i}^{\max}\right]$. As the configuration space increases, the number of qubits required for encoding will also increase, so $\Delta_{d}$ has a great impact on the quality of the solution. Next we describe how to map the collision avoidance problem to the QUBO problem.

We need to encode $D_{i}$ as a binary variable after discretizing the configuration space. In this case, we introduce a decision variable

(17) $$x_{i,\ell }=\left\{\begin{array}{cc} 1, & D_{i}=\ell \Delta_{d}\\ 0, & \;\mathrm{otherwise}\; \end{array}\right..$$

If the aircraft takes off with delay $D_{i}$, then $x_{i,\ell }$ takes the value 1; otherwise $x_{i,\ell }$ takes 0. Thus, the weighted sum of the total delays is

(18) $$f=\sum_{i=1}^{N_{c}}D_{i}=\Delta_{d}\sum_{i=1}^{N_{c}}\sum_{\ell =p_{i}+N_{i}^{\min}}^{p_{i}+N_{i}^{\max}}f\left(i\right)\cdot \ell x_{i,\ell }.$$

To transform the problem into an unconstrained problem, we define a function with penalty. We have discussed above that if (13) holds, potential conflicts can be avoided, which is also a hard constraint for collision avoidance:

(19) $$P_{con}=p_{con}\sum_{m}\sum_{\begin{array}{c} \ell ,\ell^{\prime }|\Delta_{d}\left(\ell -\ell^{\prime }\right)\in B_{m}\\ i,j\in R_{m}|i<j \end{array}}x_{i,\ell }\cdot x_{j,\ell^{\prime }},$$

where $p_{con}$ is penalized for a sufficiently large positive constant. Because each aircraft can only choose one total delay, namely

(20) $$\sum_{\ell =p_{i}+N_{i}^{\min}}^{p_{i}+N_{i}^{\max}}x_{i,\ell }=1,i\in \left[1,N_{c}\right],$$

this constraint can be transformed into the following quadratic penalty function,

(21) $$P_{en}=p_{en}\sum_{i=1}^{N_{c}}{\left(\sum_{\ell =p_{i}+N_{i}^{\min}}^{p_{i}+N_{i}^{\max}}x_{i,\ell }-1\right)}^{2},$$

where $p_{en}$ is a constant penalty. Therefore, the total cost function we need to minimize is

(22) $$Cost=f+P_{con}+P_{en}.$$

For the total cost function, we must choose an acceptable penalty. If the penalty is too large, the total cost function is only slightly perturbed, but if the penalty is too small, we will probably end up with a solution that does not work. If we let $n=\sum_{i=1}^{N_{c}}\left(N_{i}^{\max}-N_{i}^{\min}+1\right)$, then

(23) $$\sum_{i=1}^{N_{c}}\sum_{\ell =p_{i}+N_{i}^{\min}}^{p_{i}+N_{i}^{\max}}x_{i,\ell }=\sum_{k=1}^{n}x_{k},k\in \left[1,n\right],$$

that is, $x_{i,\ell }$ is equivalent to $x_{k}$, where

(24) $$\begin{array}{c} \ell \in \left[p_{i}+N_{i}^{\min},p_{i}+N_{i}^{\max}\right],\\ k\in \left(\sum_{j=1}^{i-1}\left(N_{j}^{\max}-N_{j}^{\min}+1\right),\sum_{j=1}^{i}\left(N_{j}^{\max}-N_{j}^{\min}+1\right)\right], \end{array}$$

for aircraft i≠1. When i=1, We have $\ell \in \left[p_{1}+N_{1}^{\min},p_{1}+N_{1}^{\max}\right]$ and $k\in [1,N_{1}^{\max}-N_{1}^{\min}+1]$. In particular, if the maximum system delay allowed by the aircraft and the early takeoff time are the same, they are $N^{\max}$ and $N^{\min}$, respectively. Correspondingly, for $k\in \left[1,n\right]$, we can express the serial number *i* of the aircraft and the discretized *ℓ* with *k* as follows:

(25) $$\begin{array}{c} i\left(k\right)=\left\lceil \frac{k}{N^{\max}-N^{\min}+1}\right\rceil ,\\ \ell \left(k\right)=\left(k-1\right)\;\mathrm{mod}\;\left(N^{\max}-N^{\min}+1\right)+p_{i(k)}+N^{\min}. \end{array}$$

Therefore,

(26) $$f=\Delta_{d}\sum_{k=1}^{n}f\left(i\left(k\right)\right)\cdot \ell \left(k\right)x_{k}\;,$$

(27) $$P_{\mathrm{con}\;}=\frac{p_{con}}{2}\sum_{k,k^{\prime }=1}^{n}\delta_{i\left(k\right),i\left(k^{\prime }\right)}\delta_{\ell \left(k\right),\ell \left(k^{\prime }\right)}x_{k}x_{k^{\prime }}\;,$$

(28) $$P_{\mathrm{en}\;}=p_{en}\left(\sum_{k,k^{\prime }=1}^{n}\delta_{k,k^{\prime }}x_{k}x_{k^{\prime }}-\sum_{k=1}^{n}x_{k}+N_{c}\right),$$

where

(29) $$\begin{array}{c} \delta_{k,k^{\prime }}=\left\{\begin{array}{cc} 1, & i\left(k\right)=i\left(k^{\prime }\right)\;\mathrm{and}\;k\neq k^{\prime }\\ 0, & \;\mathrm{otherwise}\; \end{array}\right.,\\ \delta_{i\left(k\right),i\left(k^{\prime }\right)}=\left\{\begin{array}{cc} 1, & i\left(k\right)i\left(k^{\prime }\right)\in R_{m}\\ 0, & \;\mathrm{otherwise}\; \end{array}\right.,\\ \delta_{\ell \left(k\right),\ell \left(k^{\prime }\right)}=\left\{\begin{array}{cc} 1, & -\frac{k-k^{\prime }}{\left|k-k^{\prime }\right|}\Delta_{d}\left(\ell \left(k\right)-\ell \left(k^{\prime }\right)\right)\in B_{m}\\ 0, & \;\mathrm{otherwise}\; \end{array}\right.. \end{array}$$

In addition, we can also remove the constant offset in formula (28) to transform it into

(30) $$\begin{array}{cc} P_{\mathrm{en}\;} & =p_{en}\left(\sum_{k,k^{\prime }=1}^{n}\delta_{k,k^{\prime }}x_{k}x_{k^{\prime }}-\sum_{k=1}^{n}x_{k}+N_{c}\right)-p_{en}\cdot N_{c}\\ \; & =p_{en}\left(\sum_{k,k^{\prime }=1}^{n}\delta_{k,k^{\prime }}x_{k}x_{k^{\prime }}-\sum_{k=1}^{n}x_{k}\right). \end{array}$$

The total cost function can also be expressed as

(31) $$Q\left(X\right)=f+P_{\mathrm{con}\;}+P_{\mathrm{en}\;}-p_{en}\cdot N_{c},X=\left(x_{1},x_{2},\ldots x_{n}\right).$$

## 3. Ising Model and Variational Quantum Algorithm

### 3.1. Ising Model and QUBO Problem

As one of the most common research areas in quantum computing and quantum annealing in the NISQ era, combinatorial optimization problems can usually be represented as quadratic unconstrained binary optimization (QUBO) problems that are easily solved by quantum computers. Researchers in combinatorial optimization have investigated the QUBO problem since the 1960s. A QUBO problem with *n* binary decision variables *x* can be expressed as

(32) $$\min_{x\in {\left\{0,1\right\}}^{n}}x^{T}Qx+b^{T}x,$$

where $Q\in R^{n\times n}$ is a matrix with symmetry, and $b\in R^{n}$ is a vector. Because the binary variable satisfies $x_{i}=x_{i}^{2}$, *b* can be added to the diagonal of Q. The symmetry of the matrix Q can always be maintained. The QUBO model has become one of the crucial elements in the NISQ era of combinatorial optimization problem solutions due to its characteristics and suitability for the Ising model [15,21]. In the beginning, the Ising model was a mathematical representation of ferromagnetism within statistical mechanics. The model is made up of several discrete variables that were initially described as the direction of the microscopic magnetic momentum associated with the atom’s “spin”, which can be in two states of +1 or −1. Therefore, the QUBO problem and the Ising model are highly correlated, and an Ising model

(33) $$\min_{z\in {\left\{-1,+1\right\}}^{n}}z^{T}Jz+h^{T}z$$

can be obtained by transforming the QUBO problem (32) through a simple mapping as follows:

(34) $$x_{i}=\frac{1+z_{i}}{2},z_{i}\in \left\{-1,+1\right\}.$$

For a quantum system containing *n* qubits, we can convert the Ising model (33) into the Hamiltonian after using the Pauli Z-matrix $\sigma_{Z}^{i}$ to replace $z_{i}$ [22]. Specifically, we can consider $z_{i}$ as acting $\sigma_{Z}^{i}$ on the ith qubit, and similarly $z_{i}\cdot z_{j}$ can be expressed using the tensor product $\sigma_{Z}^{i}⊗\sigma_{Z}^{j}$. The eigenvalue of $\sigma_{Z}^{i}$ is +1 and −1, which corresponds to the spin direction of the Ising model (33).

### 3.2. Variational Quantum Eigensolver (VQE)

The solution of the ground state *E* of a certain Hamiltonian can usually be solved by VQE, which is the first variational quantum algorithm that was proposed [14] and originally applied to the estimation of the energy of molecular ground states in quantum chemistry. In VQE, according to the Rayleigh–Ritz principle, given a Hamiltonian *H* whose minimum eigenvalue $\lambda_{\min}$ and ground state $|\psi_{min}\rangle$ are unknown, then

(35) $$\lambda_{\min}\leq \lambda_{\theta }=\langle \psi \left(\theta \right)|H|\psi \left(\theta \right)\rangle ,$$

where $|\psi \left(\theta \right)\rangle =U\left(\theta \right)|\psi_{0}\rangle$ is the result of the initial state $|\psi_{0}\rangle$ after the parameterized quantum circuit U(θ), which represents the eigenstate corresponding to $\lambda_{\theta }$, and |ψ(θ)〉 is an estimate of $|\psi_{min}\rangle$. The cost function C(θ) is defined as:

(36) C(θ)=〈ψ(θ)|H|ψ(θ)〉.

**Figure 2:**
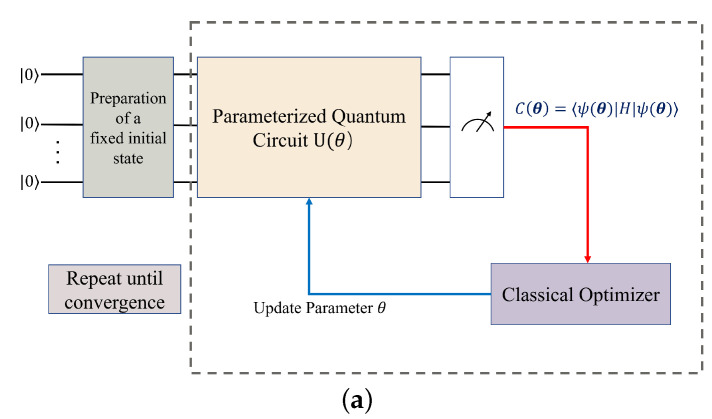

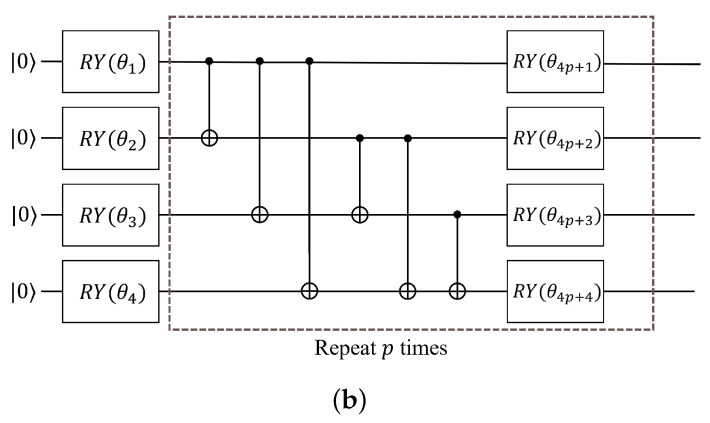
(**a**) A VQE circuit diagram. (**b**) An example of a hardware efficient ansatz for a *p*-layer with four qubits.

As shown in Figure 2a, we use the cost function C(θ) to optimize the parameter θ through a classical optimizer and continue to iterate until convergence is reached. Specifically, the parameterized quantum circuits (PQC) we use in this paper are the commonly used hardware efficient ansatz, which is able to somewhat reduce the circuit depth required to implement U(θ) [23,24]. For a quantum circuit consisting of *n* qubits, we first apply the RY-gate once to each qubit, then apply the CNOT-gate (*i* controls *j*) to all qubits satisfying i<j, and finally apply the R-gate again to each qubit, repeating the last two steps *p* times, where *p* is the number of layers. We also plotted a quantum circuit of a p-layer PQC containing four qubits (Figure 2b).

### 3.3. Quantum Approximate Optimization Algorithms

QAOA is another variational quantum algorithm proposed after VQE [5], which was influenced by quantum adiabatic algorithms and initially utilized to approximately solve combinatorial problems [25,26,27]. The combinatorial optimization problem is defined as *n* binary strings $x=\left(x_{1},\cdots ,x_{n}\right)$, and the goal of the problem is to maximize or minimize the classical objective function f(x). By transforming the classical binary variable $x_{i}$ into the Pauli spin −1/2 operator $\sigma_{i}^{z}$, QAOA maps f(x) to the problem Hamiltonian $H_{P}$. QAOA follows the ground state evolution process as

(37) $$H\left(t\right)=\left(1-t\right)H_{M}+tH_{P},t\in \left[0,1\right].$$

Usually, the problem Hamiltonian $H_{P}$ can also be expressed in the form of an Ising Hamiltonian

(38) $$H_{P}=\sum_{i=1}^{n}h_{i}\sigma_{Z}^{i}+\sum_{i,j=1}^{n}J_{ij}\sigma_{Z}^{i}⊗\sigma_{Z}^{j},$$

and the mixer Hamiltonian is defined as

(39) $$H_{M}=\sum_{i=1}^{n}\sigma_{i}^{x},$$

where $h_{i},J_{ij}$ are real coefficients, and $\sigma_{i}^{z}$ and $\sigma_{i}^{x}$ are the Pauli-Z/X operators applied to the *i*-th qubit, respectively.

**Figure 3:**
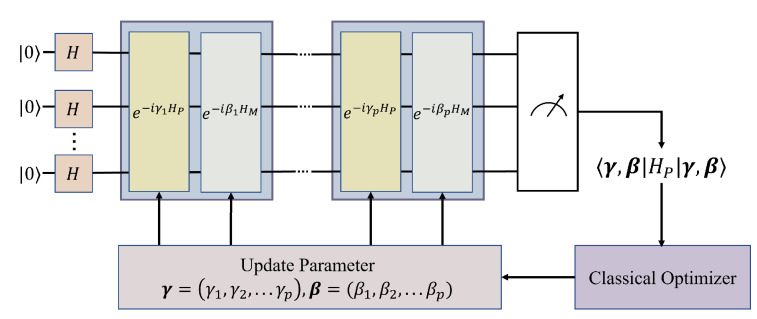
QAOA circuit diagram.

What is used in QAOA is an alternating structure ansatz, often referred to as the quantum alternation operator ansatz [28]; the adiabatic evolution is replaced by *p* alternate time propagations between $H_{P}$ and $H_{M}$. As shown in Figure 3, in the *p*-layer QAOA, the initial state is ${|+\rangle }^{⊗n}$, the evolution time interval is treated as a variational parameter, i.e., θ={γ,β}, and the two Hamiltonians are applied alternately *p* times.The variational form of QAOA is defined as

(40) $$U\left(\beta ,\gamma \right)=\left[\prod_{i=1}^{p}U_{P}\left(\beta_{i}\right)U_{M}\left(\gamma_{i}\right)\right]H^{⊗n},i\in 1,2,\ldots ,p,$$

where

(41) $$\begin{array}{cc} \; & U_{P}\left(\gamma_{i}\right)=e^{-i\gamma_{i}H_{P}}=e^{-i\gamma_{i}\left(\sum_{i=1}^{n}h_{i}\sigma_{Z}^{i}+\sum_{i,j=1}^{n}J_{ij}\sigma_{Z}^{i}⊗\sigma_{Z}^{j}\right)},\\ \; & U_{M}\left(\beta_{i}\right)=e^{-i\beta_{i}H_{M}}=e^{-i\beta_{i}\sum_{i=1}^{n}\sigma_{X}^{i}}, \end{array}$$

The quantum state generated after ansatz is

(42) |γ,β〉=U(γ,β)|0〉.

Similarly, the cost function of QAOA is defined as:

(43) $$C\left(\gamma ,\beta \right)=\langle \gamma ,\beta \left|H_{P}\right|\gamma .\beta \rangle .$$

The evaluation of the cost function is done by repeatedly measuring |γ,β〉 on a quantum computer and then optimizing and adjusting {γ,β} with the help of a classical optimizer until an acceptable {γ,β} is found.

### 3.4. Conditional Value-at-Risk

For either VQE or QAOA, we cannot directly obtain the expected value of the cost function (Equations (36) and (43)). Taking VQE as an example, we cannot get all information of |ψ(θ)〉 by just one measurement. However, we can obtain an estimate of the cost function by multiple measurements. Specifically, we can perform one measurement for *n*-qubits and get a measurement sample $q_{0}q_{1}\ldots q_{n-1}$. For this sample of measurements, we can write quantum states $|\psi_{k}\left(\theta \right)\rangle =|q_{0}q_{1}\ldots q_{n-1}\rangle$ and then calculate $E_{k}\left(\theta \right)=\langle \psi_{k}\left(\theta \right)|H|\psi_{k}\left(\theta \right)\rangle$. Then, the whole sample after *K* times measurement can be expressed as $E_{k}\left(\theta \right)\left(k=1,\ldots ,K\right)$, and the expected value of Equation (36) is the mean value of the sample

(44) $$\frac{1}{K}\sum_{k=1}^{K}E_{k}\left(\theta \right).$$

To improve the efficiency of VQE and QAOA, we attempt to use CVaR as their objective function [4]. Given a cumulative density function F(X) of a random variable *X*, the CVaR of *X* is defined as the expected value of X when $X<F^{-1}\left(\alpha \right)$ when the confidence interval is α, that is

(45) $${\mathrm{CVaR}}_{\alpha }\left(X\right)=E\left[X|X\leq F_{X}^{-1}\left(\alpha \right)\right],\alpha \in \left[0,1\right].$$

Shortly, CVaR is the expected value on the left-hand side of the distribution of *X*. Suppose that results of *K* times measurement are arranged in increasing order from smallest to largest as $\left\{E_{1},\ldots ,E_{K}\right\}$, then the corresponding $CVaR_{\alpha }$ is

(46) $$CVaR_{\alpha }=\frac{1}{\left\lceil \alpha K\right\rceil }\sum_{k=1}^{\left\lceil \alpha K\right\rceil }E_{k}.$$

The value of CVaR is the mean of the sample, which is the standard objective function when α=1.

## 4. Result

**Figure 4:**
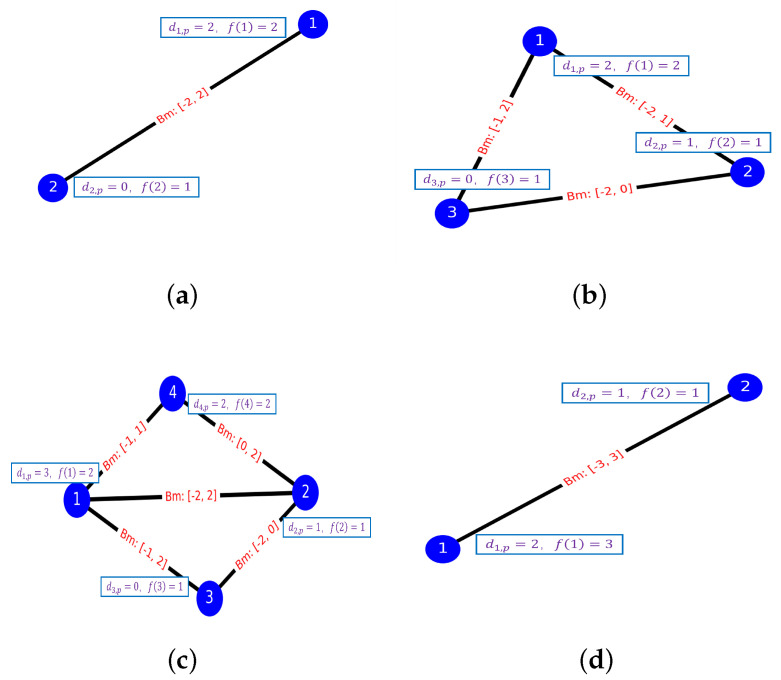
(**a**) The aircraft conflict information of (2,1) with $N^{max}=2,N^{min}=0$. (**b**) The aircraft conflict information of (3,3). (**c**) The aircraft conflict information of (4,5). (**d**) The aircraft conflict information of (2,1) with $N^{max}=2,N^{min}=-2$. The $d_{i,p}$ and f(i), respectively, represents the pre-takeoff preparation time and weight of flight i(i=1,2,3,4).

**Figure 5:**
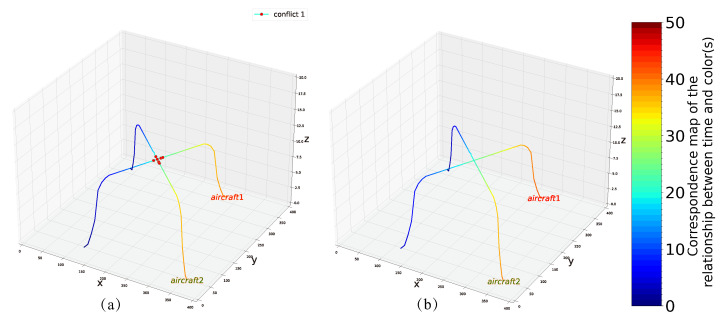
(**a**) Initial planning route for example 1. (**b**) The optimized conflict-free route in example 1.

In this section, we study four examples: (2,1) with $N^{max}=2,N^{min}=0$ (Figure 4a), (3,3) (Figure 4b), (4,5) (Figure 4c), and (2,1) with $N^{max}=2,N^{min}=-2$ (Figure 4d), where (n,k) denotes *k* pairs of conflict among *n* aircraft. We use the qasm simulator of IBM’s qiskit to solve the collision avoidance problem by VQE and QAOA. We set $\Delta_{d}=1,p_{con}=p_{en}=12$. In the first three examples, the allowable scheduling delay of each flight is set as $N^{max}=2,N^{min}=0$, so the number of qubits required is $n\cdot (N^{max}+1)$. For the last example, we set $N^{max}=2$ and $N^{min}=-2$, and the number of qubits required is $n\cdot (N^{max}-N^{min}+1)$.

In the solution process, first we encode the problem in the form of Equation (31), then map to get the Ising Hamiltonian $H_{P}$, and then use VQE or QAOA to find the ground state. We use the gradient-free optimizer linear approximation constrained optimization (COBYLA) optimizer to optimize the variable parameters of PQC for the solution of all four examples. For each example, we tested the performance of QAOA versus VQE for p=1,2 and $\alpha \in \left\{0.05,0.1,0.25,0.5,1\right\}$.

To compare the two variational quantum algorithms for different values of depth *p* and α, we also plot the number of iterations of the classical optimizer (COBYLA) against the value of the objective function under each example. In addition, we also plot the ground state sampling probabilities of the Hamiltonian during the iterative process:

(47) $$P_{|\psi_{g}\rangle }={\left|\langle \psi |\psi_{g}\rangle \right|}^{2}.$$

where $|\psi \rangle$ is the quantum state after the quantum circuit and $|\psi_{g}\rangle$ is the ground state of the Hamiltonian. We intend that the ground state probability $P_{|\psi_{g}\rangle }$ obtained by the algorithm should be as large as possible, or at least larger than our given α value.

### 4.1. Example 1

In the first example, we successfully solved example (2,1) shown in Figure 4a, whose route diagram is shown in Figure 5a. As this problem is relatively small in size, we use only six qubits to encode the problem. The lowest energy is −18, i.e., the minimum value of Equation (31), corresponding to X=(0,1,0,1,0,0), which is the optimal solution to the problem, and the total delay for each aircraft is $D_{1}=3,D_{2}=0$. In Figure 5b, we show the optimized route corresponding to the optimal solution.

### 4.2. Example 2

**Figure 6:**
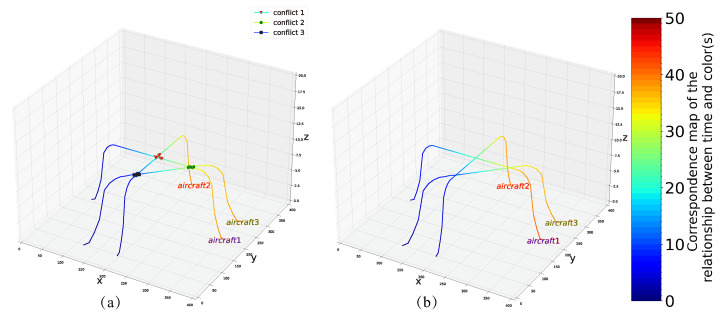
(**a**) Initial planning route for example 2. (**b**) The optimized conflict-free route in example 2.

In the second test, we try to solve example (3,3) (as shown in Figure 4b), corresponding to the route in Figure 6a, where we use nine qubits to encode the problem. Finally, we get the optimized route corresponding to the optimal solution (Figure 6b), when X=(0,1,0,1,0,0,1,0,0), the minimum energy is −29 and the total delay for each aircraft is $D_{1}=3,D_{2}=1,D_{3}=0$.

### 4.3. Example 3

**Figure 7:**
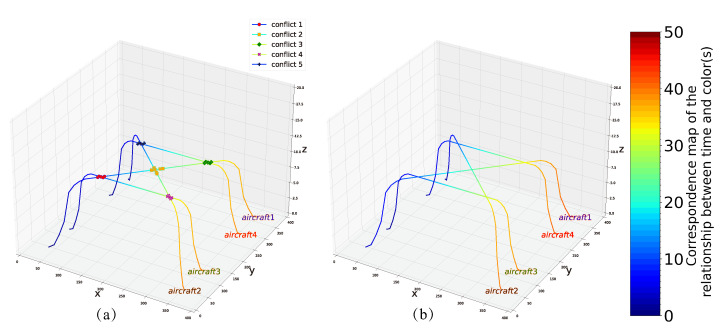
(**a**) Initial planning route for example 3. (**b**) The optimized conflict-free route in example 3.

Here, we use 12 qubits to solve the collision example of four aircraft containing five pairs of conflict, corresponding to the details of the conflicts and the routes shown in Figure 4c and Figure 7a. We obtain a minimum energy of −35, the optimal solution is X=(0,1,0,1,0,0,1,0, 0,1,0,0), and the total delay time of the four aircraft are $D_{1}=3,$ $D_{2}=1,D_{3}=0,D_{4}=2$ (the optimized routes are shown in Figure 7b).

### 4.4. Example 4

**Figure 8:**
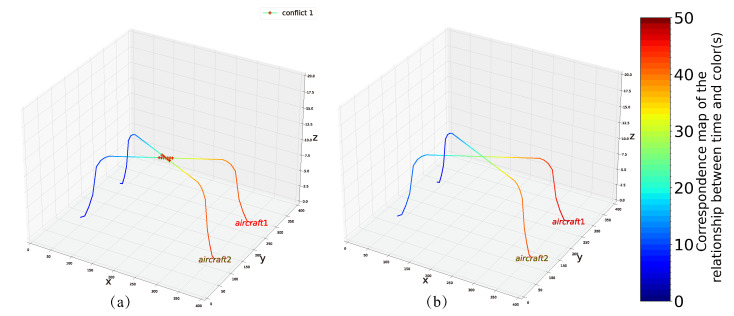
(**a**) Initial planning route for example 4. (**b**) The optimized conflict-free route in example 4.

In the last example, we solve another example of the (2,1) problem using 10 qubits (details of the conflict are shown in Figure 4d). Unlike example 1, in this example we take $N^{min}=-2$ to test the availability of the model, whereas $N^{max}$ is still set as 2. The minimum energy obtained by two algorithms separately is −16 and X=[0,0,0,1,0,1,0,0,0,0,0], while the total delay of the two aircraft is $D_{1}=3$ and $D_{2}=-1$, respectively. Similarly, we have plotted the routes of the two aircraft before and after optimization (Figure 8).

In the following discussion, we write VQE and QAOA that use CVaR as the objective function (α<1) as CVaR-VQE and CVaR-QAOA, respectively.

**Figure 9:**
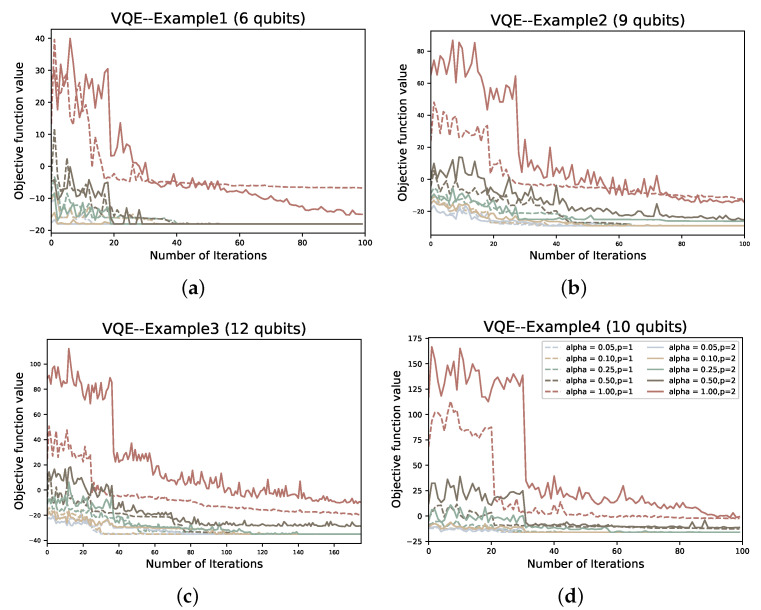
The objective function values corresponding to the number of iterations of VQE at different α and *p* in the four examples; (**a**–**d**) represent examples 1–4, respectively.

**Figure 10:**
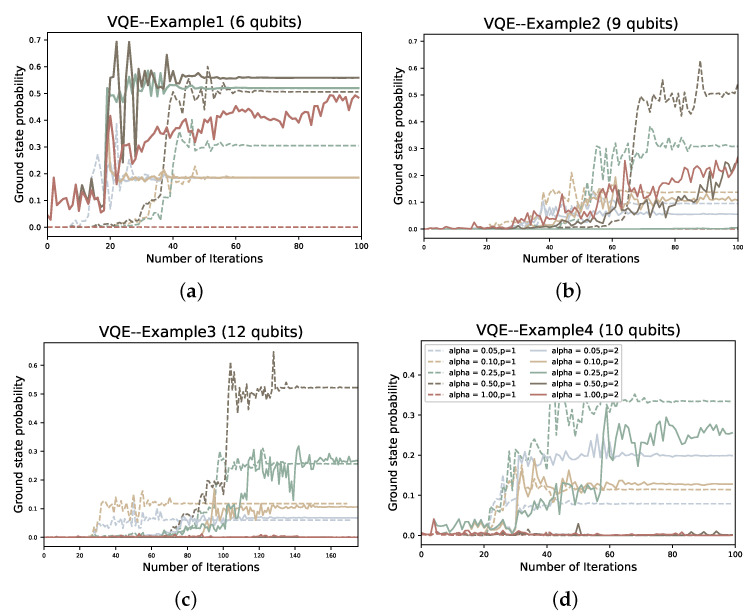
The ground state probability corresponding to the number of iterations of VQE at different α and *p* in the four examples; (**a**–**d**) represent examples 1–4, respectively.

We first compare the objective functions of the original VQE and CVaR-VQE during the iterative process. In Figure 9 and Figure 10, we plot the probability of the objective function values against the optimal solution, respectively. It is obvious from Figure 9 that when we choose α<1, the number of iterations required to converge the objective function value to the ground state energy is significantly lower than the original VQE. To take example 1, which uses six qubits, in Figure 9a, the original VQE does not reach the ground state energy until the 100th iteration, whereas even the slowest convergence, α=0.5, reaches convergence at about the 70th iteration. And as the number of qubits increases, the performance of CVaR-VQE is still superior to the original VQE, although it also decreases. For the ground state probability $P_{|\psi_{g}\rangle }$, the number of iterations required for $P_{|\psi_{g}\rangle }$ to converge increases as the α value rises, but even in example 3, which has the highest number of iterations, CVaR-VQE still has an advantage in the number of iterations required for the ground state probabilities and the objective function value to reach convergence.

**Figure 11:**
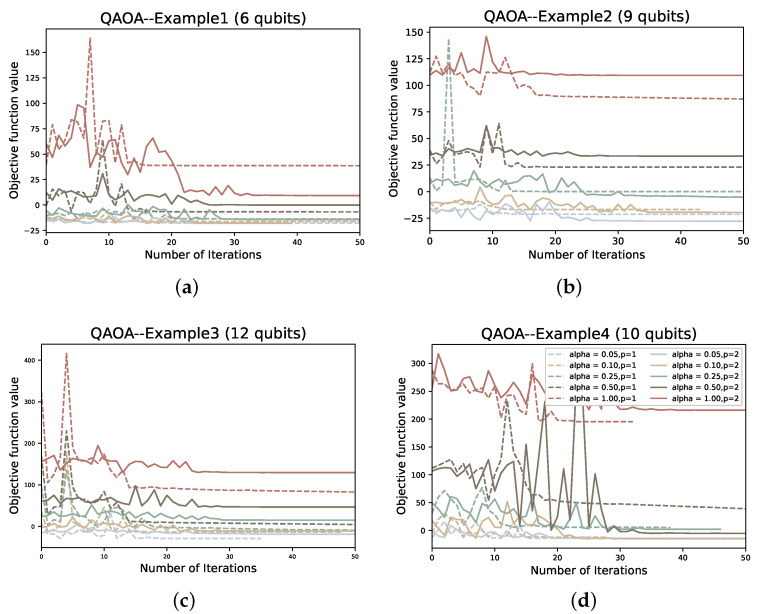
The objective function values corresponding to the number of iterations of QAOA at different α and *p* in the four examples; (**a**–**d**) represent examples 1–4, respectively.

**Figure 12:**
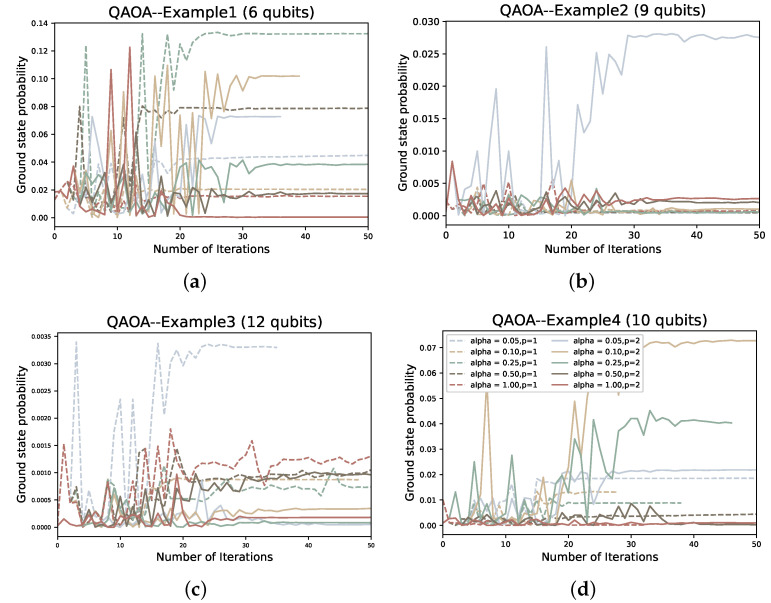
The ground state probability corresponding to the number of iterations of QAOA at different α and *p* in the four examples; (**a**–**d**) represent examples 1–4, respectively.

As the depth *p* increases and α decreases, we find that there is a certain improvement in both the objective function value and the ground state probability $P_{|\psi_{g}\rangle }$ of QAOA (as shown in Figure 11 and Figure 12). However, overall, the convergence rate of QAOA is still very slow and the ground state probability is generally very low, with the highest $P_{|\psi_{g}\rangle }$ still less than 0.15. When we optimize, most QAOA ends when the number of iterations reaches 50. Compared to the VQE, the performance of QAOA is inferior. Without considering the impact of the classical optimizer, one of the important reasons for our analysis is that the hardware efficient ansatz is used in the VQE. A VQE of depth *p* contains n(p+1) variational parameters and additional control gates, whereas the QAOA has only 2p variational parameters. Therefore, if we want to improve the performance of QAOA, increasing the depth *p* is an effective way. However, this will also inevitably lead to an increase in the solution time. Due to the current NISQ devices, it is still difficult to successfully implement quantum circuits with excessive depth. It has been shown in some previous studies that when NISQ devices run quantum algorithms, the fidelity of the quantum state prepared by the quantum program and the minimum expected value of the Hamiltonian will be affected because of the existence of noise, which makes the results have deviations [29].

**Figure 13:**
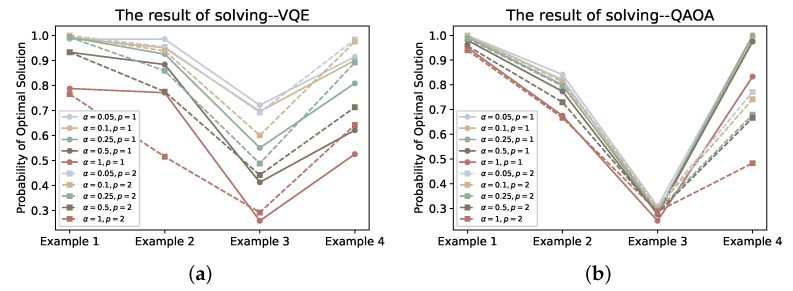
Subfigures (**a**,**b**) plot the optimal solution probabilities obtained using VQE and QAOA for different depths *p* and α values, respectively.

**Figure 14:**
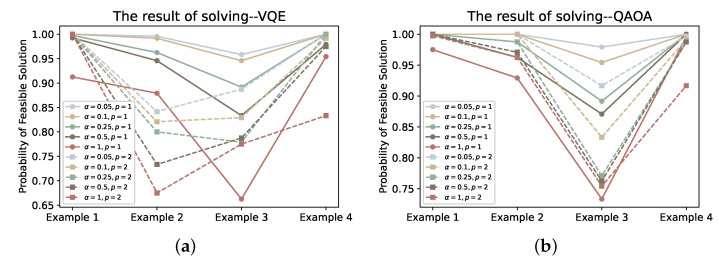
Subfigures (**a**,**b**) plot the feasible solution probabilities obtained using VQE and QAOA for different depths *p* and α values, respectively.

Finally, in order to compare the solution results, we plot the probability of obtaining the optimal solution (Figure 13) and the feasible solution (Figure 14) using VQE and QAOA in solving the four examples. From the resulting probability of optimal solution, the results of the VQE solution also confirm to some extent that the performance of VQE decreases as the α value increases. Taking the most remarkable example 3 as an example, the probability of obtaining the optimal solution even differs by 0.4 between the solution results of the original VQE and the CVaR-VQE (at α=0.05) when p=2. From the results of QAOA, the accuracy of its solution is indeed lower than that of VQE to a certain extent. In terms of the probability of feasible solutions obtained from the QAOA solution, both the application of CVaR and the increase of depth *p* have improved the performance of QAOA. Recalling our previous discussion of the existence of QAOA with small ground state probability $P_{|\psi_{g}\rangle }$ at iteration, we believe that this could be determined by the characteristics of QAOA itself—–to obtain an approximate solution to the combinatorial problem rather than an optimal solution, i.e., the quantum state be relatively flat, and this phenomenon was studied in recent work [4]. This means that the impact of the CVaR strategy we employ for QAOA is likely to be positive but relatively small. The probability of feasible solutions obtained by solving VQE and QAOA (as shown in Figure 14) also demonstrates the positive impact of CVaR on VQE and QAOA from the side.

## 5. Conclusions

In this paper, we propose a model for the aircraft collision avoidance problem and give a mapping of the model to the QUBO problem. First, the potential conflicts are formulated as mutually disjoint conflict sets. Subsequently, in order to encode the problem as a QUBO problem, we discretize the configuration space of the problem. After encoding the problem as a Hamiltonian, we use two variational quantum algorithms (VQE and QAOA) to solve the ground state of the Hamiltonian of the problem, respectively. In the experimental section, we test these two variational quantum algorithms in four different examples and compare the performance of the two algorithms under different α values and variational form depth *p*.

CVaR had a positive impact on VQA, because both CVaR-VQE and CVaR-QAOA have improved the performance of the algorithm. For the four examples we have solved, the probability of the resulting feasible solution is still approximately 70%, even for the worst solution result, and the best solution is even more than 95%. Of course, the probability of the best solution is relatively low, and the worst solution result is only approximately 30%. In conclusion, if we adjust the value of α appropriately, the probability of obtaining a feasible solution to the problem can be raised to a fairly high level. In real applications, we can obtain feasible and optimal solutions by repeatedly running the QAOA or VQE algorithms, but this also means that we must choose between the quality of the solution and the running time when solving.

The focus of this paper is on the collision avoidance problem of aircraft and the variational quantum algorithm used for this problem. For future work, we suggest that further research can be conducted in two ways.

- In model building, the main consideration of this paper is to avoid collision by extending the aircraft takeoff time. In a real situation, in order to extend the usability of the model, we can also consider making some adjustments to the flight path to avoid collision, including changing the flight speed in a specific interval and partially changing the flight path of the aircraft, etc. These strategies can be collectively referred to as maneuvering collision avoidance strategies. We can adjust the cost function of the model by mapping the maneuver cost to the time delay cost in conjunction with the actual demand.
- In the aspect of quantum computing, on the one hand, in order to improve the efficiency of model solving, we can use more efficient coding methods to reduce the number of qubits required and the depth of quantum circuits to compress the time to solve the problem when encoding the decision variables. However, this requires us to modify the mapping between the model and the QUBO problem. Second, from the algorithmic point of view, improving the classical optimizer or adopting a more efficient ansatz structure is also an effective way to improve the efficiency of the algorithm.

## Data Availability

Not applicable.

## Abbreviations

The following abbreviations are used in this manuscript:

VQA	Variational Quantum Algorithms
VQE	Variational Quantum Eigensolver
QAOA	Quantum Approximate Optimization Algorithm
QUBO	Quadratic Unconstrained Binary Optimization
ATM	Air Traffic Management
ATFM	Air Traffic Flow Management
UAV	Unmanned Aerial Vehicles
UTM	Unmanned Traffic Management
NISQ	Noisy Intermediate-Scale Quantum
CVaR	Conditional Value-at-Risk
UAM	Urban Air Mobility
NASA	National Aeronautics and Space Administration
PQC	Parameterized Quantum Circuits
